# Synthesis, Characterization, Antimicrobial and Antiproliferative Activity Evaluation of Cu(II), Co(II), Zn(II), Ni(II) and Pt(II) Complexes with Isoniazid-Derived Compound

**DOI:** 10.3390/molecules22040650

**Published:** 2017-04-19

**Authors:** Elena Pahonțu, Diana-Carolina Ilieș, Sergiu Shova, Camelia Oprean, Virgil Păunescu, Octavian Tudorel Olaru, Flavian Ștefan Rădulescu, Aurelian Gulea, Tudor Roșu, Doina Drăgănescu

**Affiliations:** 1 Inorganic Chemistry Department, Faculty of Pharmacy, University of Medicine and Pharmacy “Carol Davila”, 6 Traian Vuia Street, 020956 Bucharest, Romania; 2 Organic Chemistry Department, Faculty of Pharmacy, University of Medicine and Pharmacy “Carol Davila”, 6 Traian Vuia Street, 020956 Bucharest, Romania; 3 Institute of Macromolecular Chemistry “Petru Poni”, 41A Grigore Ghica Voda Alley, 700487 Iasi, Romania; shova@icmpp.ro; 4 Environmental and Food Chemistry Department, Faculty of Pharmacy, University of Medicine and Pharmacy “Victor Babeş”, 2nd Eftimie Murgu Sq., 300041 Timişoara, Romania; camelia.sass5@gmail.com; 5 “Pius Brinzeu” Timișoara County Emergency Clinical Hospital, Oncogen Institute, 156 Liviu Rebreanu, 300723 Timişoara, Romania; genostem@yahoo.com; 6 Functional Sciences Department, Faculty of Medicine, University of Medicine and Pharmacy “Victor Babeş”, 2 Eftimie Murgu Square, 300041 Timişoara, Romania; 7 Pharmaceutical Botany and Cell Biology Department, Faculty of Pharmacy, University of Medicine and Pharmacy “Carol Davila”, 6 Traian Vuia Street, 020956 Bucharest, Romania; octav_olaru2002@yahoo.com; 8 Pharmaceutical Biotechnology Department, Faculty of Pharmacy, University of Medicine and Pharmacy “Carol Davila”, 6 Traian Vuia Street, 020956 Bucharest, Romania; flavian_stefan@yahoo.com; 9 Coordination Chemistry Department, Moldova State University, 60 Mateevici Street, 2009 Chisinau, Moldova; dociu1946@yahoo.com; 10 Inorganic Chemistry Department, Faculty of Chemistry, University of Bucharest, 23 Dumbrava Rosie Street, 020462 Bucharest, Romania; t_rosu0101@yahoo.com; 11 Pharmaceutical Physics Department, Faculty of Pharmacy, University of and Pharmacy “Carol Davila”, 6 Traian Vuia Street, 020956 Bucharest, Romania; doinadraganescu@yahoo.com

**Keywords:** copper(II), cobalt(II), zinc(II), nickel(II) and platinum(II) complexes, antimicrobial activity, human breast SKBR-3 and MCF-7, human melanoma A375, lung adenocarcinoma cell NCI-H1573

## Abstract

Hydrazone complexes of Cu(II), Co(II), Zn(II), Ni(II) and Pt(II) with *N*-isonicotinoyl-*N*′-(3-metoxy-2 hydroxybenzaldehyde)-hydrazone (**HL**) were synthesized and characterized by different physico-chemical techniques including elemental and thermal analysis, magnetic susceptibility measurements, molar electric conductivity, as well as IR (infrared), ^1^H-NMR and ^13^C-NMR (hydrogen and carbon nuclear magnetic resonance, UV-Vis (ultraviolet-visible), FAB (fast atom bombardment), EPR (electron paramagnetic resonance), and mass spectroscopies. The crystal structure of ligand was determined by single crystal X-ray diffraction studies. Spectral data showed that hydrazone behaves as an ONO tridentate ligand through the azomethine nitrogen, phenolate and keto oxygen atoms. For the copper(II) complexes, metal–ligand bonding parameters were evaluated from the EPR spectra. These parameters indicate the presence of in-plane π bonding. In addition, the f values of complexes **1**–**4** indicate small distortion from planarity. The effect of these complexes on proliferation of human breast cancer (MCF-7 and SKBR-3), human melanoma (A375), lung adenocarcinoma cells (NCI-H1573) and their antibacterial activity against *Escherichia coli*, *Klebsiella pneumoniae*, *Staphylococcus aureus* and *Candida albicans* strains were studied and compared with those of free ligand. The ligand and complexes **1**–**3** showed significant antimicrobial activity against the Gram-positive bacteria *Staphylococcus aureus* and *Candida albicans* in comparison to the control drugs. The complexes **2**–**4** could be potential antitumor agents, leading to a significant improvement of the cytotoxic activity when compared with **HL**.

## 1. Introduction

Since its discovery in 1921, isoniazid has enjoyed a lot of attention due to its antituberculostatic, antidepressant and antibacterial properties [1]. The discovery of these properties has enabled further research on isoniazid and its derivatives [2].

It has since been found that isoniazid-type hydrazone derivatives are more efficient and less hepatotoxic than isoniazid, due to blockage of the terminal amino group [3].

The interest in the study of hydrazones possessing donor properties has increased in the last years, and research has shown that their pharmacological activity is due to their ability to form chelates with physiological metal ions [4]. Therefore, a great number of hydrazones as well as their corresponding complexes have antibacterial, antifungal, antiviral, antioxidative and antitumoral activities [5,6,7,8,9,10]. Also, ligands derived from salicylaldehyde represent an important class of compounds due to their ability to be used in various fields [11]. Coordination geometry, type of donor atom present in ligands, the metal ion type and its valence play a major role in the biological effect of these complexes [12,13,14].

In this article, we present the synthesis and characterization of a new hydrazone derived from isoniazid (**HL**) and also the study of the chelating behavior of this ligand towards the transition metal ion copper(II), cobalt(II), zinc(II), nickel(II) and platinum(II).

In view of their possible biological properties, we evaluated the antimicrobial activity of the free ligand and its metal complexes using the paper disc diffusion method [15] (for the qualitative determination) and the serial dilutions in liquid broth method [16] (for determination of minimum inhibitory concentration—MIC). For the toxicity testing of the compounds, the *Daphnia magna* bioassay was used. The complexes and ligand were also tested for their in vitro antiproliferative activities using four different cancer cell lines: MCF-7, SKBR-3, A375 and NCI-H1573.

## 2. Results and Discussion

### 2.1. Chemistry

The hydrazone (**HL**) was synthesized in DMSO-CH_3_OH with good yield. The structure of formed ligand was established by IR (infrared), UV–Vis (ultraviolet-visible), ^1^H-NMR, ^13^C-NMR (hydrogen and carbon nuclear magnetic resonance) and mass spectroscopies, in addition to X-ray crystallography. All complexes were synthesized by direct reaction of the inorganic salt solutions with the ligand solution using a molar ratio of 1:1 (M:L). The obtained complexes are microcrystalline solids which are stable in air. The melting point values are greater than 291 °C. The synthesis of the complexes is reproducible and the hydrazone coordinates as a tridentate ligand through the azomethine nitrogen, the oxygen atom of the isoniazid moiety and the phenolic oxygen atom.

The molar conductivity values (2–18 ohm^−1^·cm^2^·mol^−1^) of complexes **1**–**8** in 10^−3^ M DMF solution show that they are non-electrolytes [17].

Thermal analyses data reveal that compounds **2**, **5**–**8** are not hydrated.

**Scheme 1:**
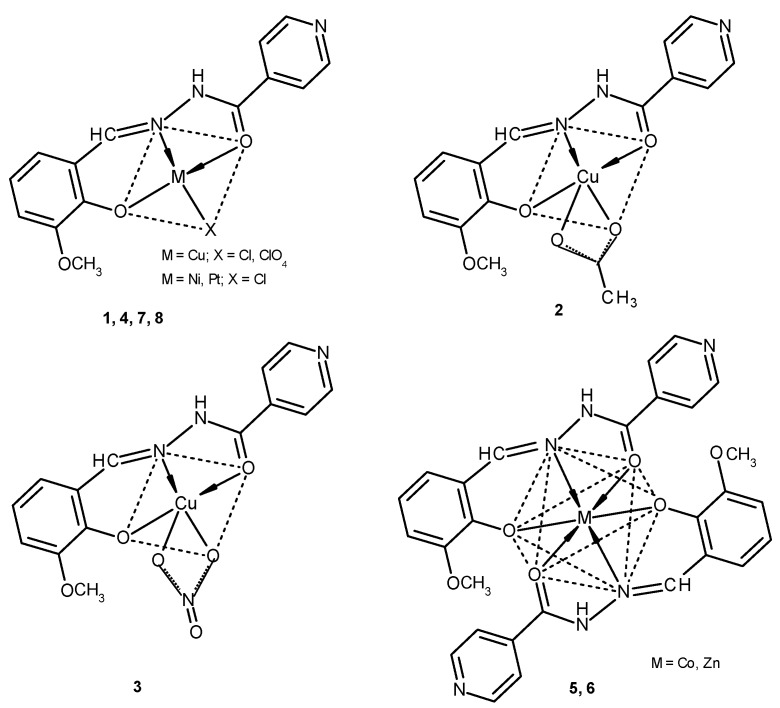
Proposed structures of the metal complexes.

**Figure 1:**
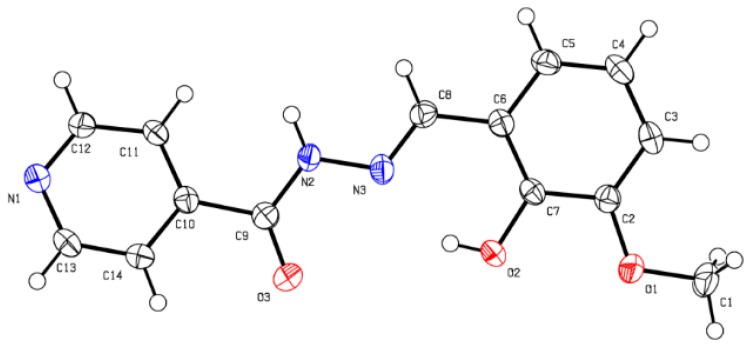
Perspective view of the hydrazone (**HL**) molecule, along with atom numbering.

The elemental analyses data of the hydrazone and their complexes (see Experimental section) are compatible with structure of the ligand shown in Figure 1 and with the proposed structures of the complexes shown in Scheme 1.

#### 2.1.1. X-ray Crystallography

Although we used a new method of synthesis for the ligand, crystal structure (Figure 1) and crystallographic data coincide (Supplementary Materials Table S1) with those obtained previously [18].

#### 2.1.2. Infrared Spectra and Coordination Mode

The IR spectra of the complexes are compared with that of the free ligand to determine the changes that might occur during complex formation. The IR spectrum of the ligand exhibits a strong band at 1647 cm^−1^ assignable to ʋ(C=N). In the spectra of the respective complexes, this band disappears, thereby suggesting the coordination of the azomethine nitrogen to the metal center [19].

The phenolic ʋ(C–O) stretching vibration in the free hydrazone is observed at 1157 cm^−1^, which is shifted by 10–19 cm^−1^ towards lower wavenumbers or disappears in the complexes, thus indicating coordination of the phenolic oxygen to the metallic ions [20]. Also, stretching frequencies of isoniazid moiety, ʋ(C=O), due to carbonyl group at 1689 cm^−1^ in the free ligand is shifted, in the IR spectra of the complexes, with 11–20 cm^−1^, to lower wave numbers. This indicates that the carbonyl oxygen is involved in the coordination [21].

The acetato complex **2** has two strong bands at 1529 and 1434 cm^−1^ corresponding to ʋ_as_ (COO^−^) and ʋ_s_ (COO^−^) with a difference between frequencies of 95 cm^−1^. This difference confirms the bidentate nature of the coordinated acetate group [22].

The nitrato complex **3** has two bands at 1530 and 1214 cm^−1^ corresponding to ʋ_5_ and ʋ_1_, with a separation of 316 cm^−1^ and a medium band at 1032 cm^−1^ assignment to ʋ_2_ of the nitrato group. These values indicating the presence of a terminal bidentate nitrato group [23].

The perchlorate complex **4** shows a band at 1115 cm^−1^ assignable to ʋ_3_(ClO_4_^−^) and a strong band at 1083 cm^−1^ assignable to ʋ_4_(ClO_4_^−^). The splitting of this band in two components indicates the presence of a monodentate perchlorate group [24].

In the IR spectra of complexes **1**, **3** and **4** a considerable peak observed in the 3450–3420 cm^−1^ range supports the presence of ʋ(H_2_O) in the complexes [25,26].

#### 2.1.3. ^1^H-NMR and ^13^C-NMR (Hydrogen and Carbon Nuclear Magnetic Resonance) Spectra 

The ^1^H-NMR (DMSO-*d*_6_) spectra of diamagnetic Zn(II) and Pt(II) complexes showed the disappearance of the signal due to the OH proton indicating the deprotonation of this group. The signal due to the =N-NH proton remained unchanged as observed in the free ligand. The signals of the HC=N protons shift downfield in complexes and appeared at 8.90 and 9.20 ppm. In the ^13^C-NMR (DMSO-*d*_6_) spectra of the two complexes it was observed that the C=O carbon atoms’ signal shifts downfield and appears at 161.56 and 161.72 ppm, respectively. This information indicates the coordination of the metal center to the azomethine nitrogen, phenolate and keto oxygen atoms [27].

#### 2.1.4. Electronic Spectra and Magnetic Studies

The geometry of the metal complexes has been deduced from the electronic spectra of the complexes. The tentative assignments of the significant electronic spectral bands of ligand and of complexes along with the values for the magnetic moment are presented in Table 1. The electronic spectra in the polycrystalline state of **HL** show the intraligand absorption maxima corresponding to π→π* and n→π* transitions: 30,300 and 26,670 cm^−1^. In the spectra of the complexes these bands are shifted to lower energies.

The spectra of complexes **1** and **4** exhibit d–d bands (Figure S1) corresponding to the transitions specific to the square-planar complexes with $d_{x}^{2}-d_{y}^{2}\;$ ground state [28]. The magnetic moment values (1.46 and 1.56 BM) for complexes **1** and **4** are indicative of square-planar geometry [29].

Likewise, the electronic spectra of the Cu(II) complexes **2** and **3** suggest the typical axial behavior for a square-pyramidal geometry. Also, the room-temperature magnetic moment values (1.73, 1.78 BM) indicate the presence of an unpaired electron on Cu(II) ion in an ideal square-pyramidal environment [30].

The electronic spectrum of complex **5** displayed two bands assignable to ^4^T_1g_(F)→^4^A_2g_(F) and ^4^T_1g_(F)→^4^T_1g_(P) transitions, respectively consistent with high spin octahedral Co(II) [31]. The magnetic moment value and the brown color for this complex are more consistent with octahedral stereochemistry [32].

The electronic spectrum of the nickel(II) complex **7** shows two spin-allowed bands at 11,420 and 15,620 cm^−1^, respectively. These absorption bands may be assigned to the ^3^A_2g_(F)→^3^T_1g_(F) and ^3^A_2g_(F)→^3^T_1g_(P) transitions, respectively, and reflect Ni(II) d^8^ ions in a square-planar environment [33]. The magnetic moment value (3.46 BM) corresponds to two unpaired electrons per metal ion.

For complex **8**, the electronic spectrum showed a square-planar geometry for the platinum ion. The very intense band at about 23,260 cm^−1^ is assignable to a combination of metal-ligand charge transfer and d–d bands [34].

#### 2.1.5. Mass Spectra

The FAB mass spectra of Cu(II), Co(II), Ni(II), Zn(II) and Pt(II) complexes with hydrazone **HL** have been recorded (Table S2). The mass spectrum of ligand showing a peak at *m*/*z* = 272.1 corresponds to the molecular ion [M]^+^ (Figure S2).

The molecular ion [M]^+^ peaks obtained from complexes are as follow: *m*/*z* = 407.1 (**1**), *m*/*z* = 394.9 (**2**), *m*/*z* = 411.1 (**3**), *m*/*z* = 452.2 (**4**), *m*/*z* = 604 (**5**), *m*/*z* = 605.1 (**6**), *m*/*z* = 365.1 (**7**), *m*/*z* = 501.2 (**8**). The data obtained are in good agreement with the proposed molecular formula for complexes **1**–**8**. The FAB mass spectra of these complexes show peaks assignable to various fragments arising from the thermal cleavage of the complexes.

#### 2.1.6. Thermal Decomposition

All complexes studied were investigated by thermogravimetry analysis. For complexes **1** and **3** the thermogravimetric analysis show a weight loss in the range 30–120 °C. This mass loss corresponds to the elimination of two water molecules (**1**) and one molecule of water of dehydration for complex **3**, respectively. The second weight loss steps correspond to the release of small coordinated anions: Cl^−^, OAc^−^, NO_3_^−^. The other weight loss refers to the decomposition of the ligand. The final residue for complexes (**1**–**3**) was analyzed by IR spectroscopy, which confirms the formation of CuO (Figure S3).

#### 2.1.7. Electron Paramagnetic Resonance (EPR) Spectra

The electron paramagnetic resonance (EPR) spectra of the copper(II) complexes were recorded in the polycrystalline state and DMSO solution to provide information about the coordination environment around the copper(II) ion.

**Figure 2:**
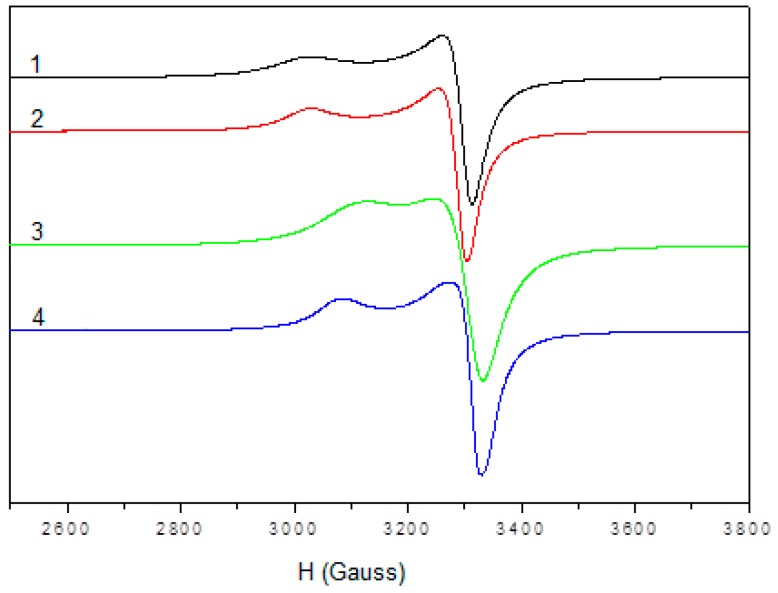
Electron paramagnetic resonance (EPR) spectra of complexes **1**–**4** in the polycristalline state at 298 K.

The EPR spectral assignments of the Cu(II) complexes **1**–**4** and orbital reduction parameters in the polycrystalline state at 298 K and in dimethyl sulfoxide (DMSO) solution at 298 K and 77 K are presented in Table 2. The spectra of complexes **1**–**4** show typical axial behavior with slightly different $g_{\|}$ and g⊥values (Figure 2). In these Cu(II) complexes, tensor values of $g_{\|}$ > $g_{⊥}>$ 2.002 are consistent with a $d_{x}^{2}-d_{y}^{2}$ ground state [35,36]. Also, a signal at half field (1600 G), specifically copper dimers, was not observed. The geometric parameter G is a measure of the exchange interaction between copper centers in a polycrystalline compound. It is calculated using the equation G = (g‖ − 2.0023)/(g⊥ − 2.0023) for axial spectra. G values less than 4.0 indicate a small exchange coupling (complex **3**). If G is higher than 4.0, the exchange interaction is negligible (complexes **1**, **2**, **4**) [37].

**Figure 3:**
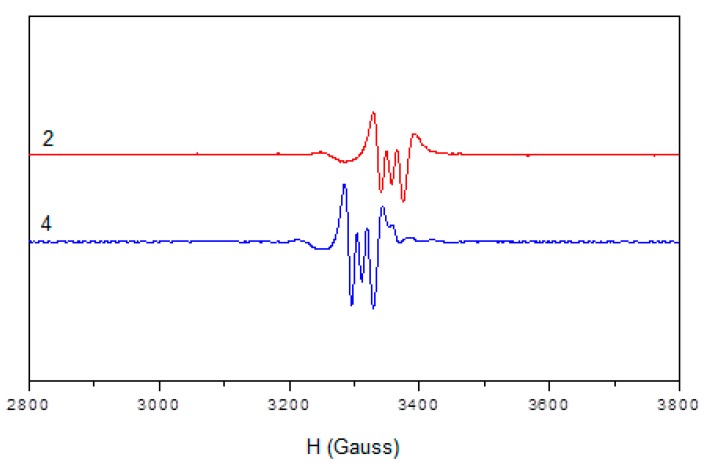
EPR spectra of complexes **2** and **4** in dimethyl sulfoxide (DMSO) solution at 298 K (second derivative).

Spectra of complexes **1**–**4** show three nitrogen superhyperfine lines in the perpendicular component (Figure 3).

The EPR parameters gǁ, g⊥, Aǁ and the energies of the d–d transition were used to evaluate the bonding parameters α^2^, β^2^ and δ^2^, which may be regarded as measures of the covalency of the in-plane σ bonds, in-plane π bonds and out-of-plane π bonds [38]. The orbital reduction factors Kǁ = α^2^β^2^ and K⊥ = α^2^δ^2^, were calculated using expressions reported elsewhere [39]. In all complexes, the Kǁ, K⊥ values, are in agreement with the relation Kǁ < K⊥ which indicates the presence of in-plane π bonding [40].

The empirical factor f = gǁ/Aǁ cm^−1^ is an index of tetragonal distortion. Values of this factor may vary from 105 to 135 for small to extreme distortions in square planar complexes, dependingon the nature of the coordinated atoms [41]. The f values of complexes **1**–**4** are found to be in the range 121–127, indicating small distortion from planarity.

### 2.2. Solubility Tests

The compounds displayed a reduced solubility in compendial buffers, especially in the medium simulating the small intestine (pH = 6.8) where quantifiable levels were obtained only for the organic ligand **HL** (Table 3). In most cases, acidic conditions generated the highest solubility values, possibly by protonation. Considering a potential administration on oral pathway, it seems likely that in the proximal regions of gastrointestinal tract, the solubility decreases, probably due to the weak basic properties of the exposed functional groups. The organic ligand is the only structure which preserves the free groups, probably due to involvement in the development of hydrogen bonding. The pH closest to the neutral region generates the less polar and, theoretically, more permeable molecular species. Notably, only one compound, i.e., complex **8**, has a molecular weight beyond the 450 cut-off value proposed by Lipinski in its mnemonic rule of five. It may be concluded that, despite the reduced water solubility, the absorption risks of the considered compounds remain low.

### 2.3. Daphnia magna Toxicity Assay

In the last decades, alternative methods of testing toxicity have been developed and used. These methods are simple, quick and economical and involve plant and invertebrate organisms [42,43,44,45]. *Daphnia magna* is a cladoceran organism used frequently along with *Artemia salina* in the cytotoxicity and biological activity evaluation of plant extracts, synthesis compounds and pollutants [46,47,48].

The toxicity of newly synthesized compounds was evaluated using the *Daphnia magna* bioassay. The crustaceans are sensitive to small quantities of metals like Cu, Ni, Zn, Co and Pt [49]. According to previous works, the most toxic is Cu, followed by Zn, Ni, Co and Pt [50]. The results of *Daphnia magna* bioassay are given in Table 4 and the lethality curves in Figure S4. The highest toxicity was exhibited by Cu^2+^ salts. Thus, after 24 h of exposure, the toxicity varies from perchlorate, chloride, nitrate and acetate. The copper acetate has a LC_50_ about 25-fold lower than the perchlorate. The toxicity of the other salts varies in the following descending order: ZnCl_2_, K_2_PtCl_4_, NiCl_2_ and CoCl_2_. After 48 h, the order of toxicity is slightly modified, yet the most toxic remained copper perchlorate and the less toxic cobalt chloride. The **HL** toxicity is moderate to high and it is constant throughout the experiment. The complexes formed by the metal ions with **HL** are less toxic than the salts, and, for (**1**) and (**8**) less toxic than both ligand and salt. The toxicity induced by **HL** at 24 h is significantly higher than that of compounds (**1**), (**3**), (**5**), and (**7**). Compound (**2**) showed a comparable toxicity, whereas compound (**6**) was slightly more toxic than **HL**. After 48 h, with the exception of (**7**), (**8**) and (**1**), all other compounds exhibited higher toxicity than **HL**. Regarding the toxicity exhibited by the precursor metal salts, compound (**6**) presents a comparable toxicity with ZnCl_2_ and (**5**) was more toxic than CoCl_2_. The other compounds showed significantly lower values of LC_50_, which correspond to a higher toxicity. Among complexes, the combination of **HL** with Cu(ClO_4_)_2_ is the less toxic, followed in ascending order by (**8**), (**1**), (**7**), (**3**), (**5**), (**2**) and (**6**) (after 24 h), and by (**7**), (**8**), (**1**), (**2**), (**3**), (**5**) and (**6**) (at 48 h). The differences between 24 h and 48 h toxicity is probably caused by the pharmacokinetic behavior.

### 2.4. Antibacterial and Antifungal Activity

All synthesized complexes and the ligand were screened for their in vitro antibacterial activity against Gram-positive bacteria (*Staphylococcus aureus*), Gram-negative bacteria (*Escherichia coli*, *Klebsiella pneumoniae*) and antifungal activity (*Candida albicans*) strains using the paper disc diffusion technique (for the qualitative determination) and the serial dilutions in liquid broth method (for determination of MIC). Furacillinum and nystatine were used as standard drugs.

Experimental results obtained from the study of antimicrobial activity (Table 5) demonstrate that complexes display bacteriostatic and bactericide activity in the concentration range 0.07–500 µg/mL towards both Gram-positive as well as Gram-negative bacteria. The remarkable activity of the ligand **HL** against Gram-positive bacteria, which is the subject of a patent application, may be due to the presence of the azomethine group which is important in elucidating the mechanism of transformation reaction in biological system. The results for antibacterial activities revealed that compounds **1**–**5**, **7** and **8** exhibited good activity against *Staphylococcus aureus*. The copper complexes **1**–**3** displayed the best activity against *Staphylococcus aureus* compared to other complexes and furacillinum.

The data concerning the study of antimycotic properties of compounds **1**–**8** show that they also display selective bacteriostatic and bactericide activity in the concentration range 0.7–250 µg/mL towards investigated fungi stem. The results show that complexes **1**, **3**, **4** have antimycotic activity against *Candida albicans*, higher than nystatine activity. The minimum inhibitory concentration (MIC) and minimum bactericide concentration (MBC) are influenced by the nature of the metal ion as well as the presence of an anion in the coordination sphere.

### 2.5. Antiproliferative Activity

**Figure 4:**
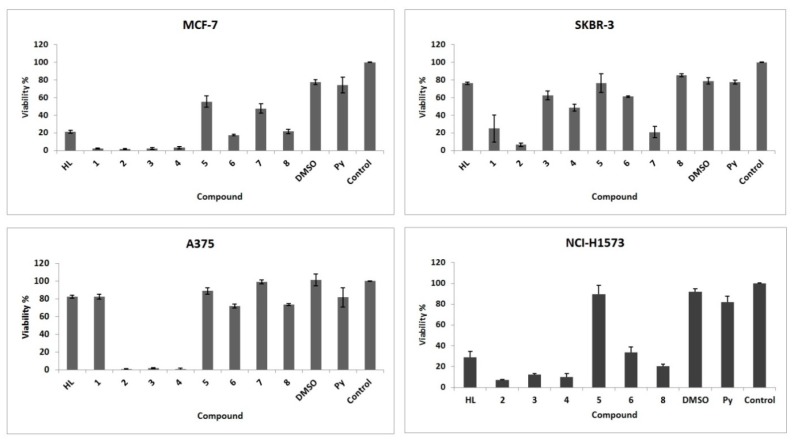
Cell viability of the MCF-7, SKBR-3, A375 and NCI-H1573 cancer cells after 48 h treatment with **HL** and the metal complexes.

Figure 4 shows the cell viability percentage of the MCF-7, SKBR-3, A375 and NCI-H1573 cancer cells after 48 h treatment with **HL** and the obtained complexes. One can notice (Table S3) that **HL** acted in a different manner on the fourth cancer cell lines. On the MCF-7 (breast) and NCI-H1573 (lung) cells, **HL** had a significant cytotoxic activity, cell viability being below 30% of the control (untreated). Not the same activity could be observed for A375 (melanoma) and SKBR-3 (breast) lines, where **HL** produced only a slight cytotoxic activity, with the same intensity that one produced by DMSO and pyridine. A significant improvement of the cytotoxic effect was observed for the compounds **2**–**4** in all four cell lines tested. No improvement of the cytotoxic effect was observed for compound **5**, while compounds **1**, **6**, **7** and **8** acted differently, depending on the cell type. Except for the NCI-H1573 cell, compound **6** showed a cytotoxic activity, while compound **1** showed an increased cytotoxic effect on both breast cancer cell lines (MCF-7 and SKBR-3). Regarding the activity of complexes **7** and **8**, a better cytotoxic effect was observed only for the MCF-7 and SKBR-3 lines in complex **8**, while for complex **7**, an inhibitory effect was observed only on SKBR-3 cells.

## 3. Experimental Section

### 3.1. General Information

Isoniazid and 2-hydroxy-3-methoxy-benzaldehyde (Sigma-Aldrich, Munich, Germany) were used as received. CuCl_2_·2H_2_O, Cu(CH_3_COO)_2_·H_2_O, Cu(NO_3_)_2_·3H_2_O, Cu(ClO_4_)_2_·6H_2_O, CoCl_2_·6H_2_O, ZnCl_2_, NiCl_2_·6H_2_O and K_2_[PtCl_4_] (Merck, Darmstadt, Germany) were used as supplied. Solvents used for the reactions were purified and dried by conventional methods [51].

Caution! Perchlorate salts of metal complexes with organic ligands are potentially explosive and should be handled with care.

The chemical elemental analysis for the determination of C, H, N was done with a Carlo-Erba LA-118 microdosimeter. The copper(II) content has been determined by atomic absorption spectroscopy with a Carl Zeiss Jena AAS 1N spectrometer. IR spectra were recorded on a Specord-M80 spectrophotometer in the 4000–400 cm^−1^ region using KBr pellets. ^1^H-NMR and ^13^C-NMR spectra were recorded at room temperature on a Bruker DRX 400 spectrometer (Billerica, MA, USA) in DMSO-*d*_6_, using TMS as the internal standard. The complexes were studied by thermogravimetry (TG) in a static air atmosphere, with a sample heating rate of 10 °C/min using a STA 6000 Perkin Elmer (Waltham, MA, USA). Electronic spectra were recorded using the JascoV-670 spectrophotometer (Tokyo, Japan), in diffuse reflectance, using MgO dilution matrices. The FAB mass spectra were recorded on JMS-AX-500 mass spectrometer (Tokyo, Japan). GCMS analysis was performed on a Shimadzu GCMS-QP5050A instrument (Shimadzu, Duisburg, Germany). EPR spectra were recorded on polycrystalline powders and DMSO solutions at room temperature and 77 K with a MiniScope MS200, Magnettech Ltd. (Berlin, Germany), X-band spectrometer (9.3–9.6 GHz), connected to a PC equipped with a 100 KHz field modulation unit. The molar conductance of the complexes in dimethylformamide solutions (10^−3^ M), at room temperature, were measured using a Consort C-533 conductivity instrument. The magnetic susceptibility measurements were done at room temperature in the polycrystalline state on a Faraday magnetic balance (homemade). Crystallographic measurements were carried out on an Oxford-Diffraction XCALIBUR E CCD diffractometer (Santa Clara, CA, USA) equipped with graphite-monochromated MoKα radiation. The single crystals were positioned at 40 mm from the detector and 197 and 273 frames were measured each for 20 and 30 s over 1° scan width for **HL**. The unit cell determination and data integration were carried out using the CrysAlis package of Oxford Diffraction (Oxfordshire, United Kingdom) [52]. The structure was solved by direct methods using Olex2 (Durham, United Kingdom) [53] software with the SHELXS structure solution program and refined by full-matrix least-squares on *F*^2^ with SHELXL-97 [54].

### 3.2. Synthesis

#### 3.2.1. Synthesis of the *N*-Isonicotinoyl-*N*′-(3-Metoxy-2 Hydroxybenzaldehyde)-Hydrazone (**HL**)

To a solution of isoniazid (0.137 g, 1 mmol) dissolved in methanol/DMSO (2:1, *v*/*v*, 15 mL) a solution of 2-hydroxy-3-methoxy-benzaldehyde (0.152 g, 1 mmol) in methanol (10 mL) was added. The resulting mixture was refluxed for 9 h at constant temperature and kept at 4 °C for six days. Then, the yellow precipitate was filtered, washed with methanol and recrystallized from methanol–ethanol (1:1, *v*/*v*). Fine yellow crystals obtained upon slow evaporation at room temperature were characterized, including single crystal X-ray diffraction.

Yield 79%; M.p. 235 °C; *Anal.* Calc. for C_14_H_13_N_3_O_3_: C, 61.99; H, 4.79; N, 15.49%. Found: C, 62.17; H, 4.57; N, 15.28%. IR (KBr, cm^−1^): υ(NH) 3201; υ(C=O) 1689; υ(C=N) 1647; υ(Ar-OH) 1157; υ(OH) 3004; ʋ(Ar–O–C_aliphatic_) 1250.

^1^H-NMR (DMSO-*d*_6_; d, ppm): 3.82 (s, 3H methyl); 6.80–7.46 (m, 7H benzene and pyridine), 8.69 (s, 1H, CH-); 7.89 (s, 1H, NH-N=). ^13^C-NMR (DMSO-*d*_6_; d, ppm): 55.92 (OCH_3_); 114.01; 119.07; 119.32; 120.41; 121.86; 122.80; 122.72; 147.20; 148.07; 148.91 (benzene and pyridine); 140.51 (C-OH); 150.17 (C=N); 161.38 (C=O).

#### 3.2.2. General Procedure for the Preparation of the Metal Complexes (**1**–**8**).

Complexes **1**–**8** were prepared by the direct reaction between the ligand and the corresponding metal salts.

*Synthesis of the complex [Cu(L)(Cl)]·2H_2_O* (**1**). To **HL** (0.271 g, 1 mmol) dissolved in 20 mL chloroform/methanol solution (3:1, *v*/*v*) CuCl_2_·2H_2_O (0.170 g, 1 mmol) dissolved in 10 mL methanol was added. The mixture was stirred for 6 h at 50 °C. The brown-colored solid was filtered, washed with hot methanol followed by ether and dried in vacuo. Yield: 81%; M.p. 292 °C; M.wt.: 405; Anal. Calc. for C_14_H_16_CuN_3_O_5_Cl: C, 41.48; H, 3.95; Cu, 15.67; N, 10.37%. Found: C, 41.67; H, 3.57; Cu, 15.39; N, 10.20%. Main IR peaks (KBr, cm^−1^): υ(NH) 3195; υ(C=O) 1670; υ(C=N)-; υ(Ar-OH) 1125; ʋ(Ar–O–C_aliphatic_) 1244. The complex is soluble in acetone, acetonitrile, DMF and DMSO, and is partially soluble in ethanol and methanol.

*Synthesis of the complex [Cu(L)(CH_3_COO)]* (**2**). Complex **2** was prepared in a similar fashion as complex **1**, using Cu(OAc)_2_·H_2_O (0.199 g, 1 mmol). Brown solid. Yield: 83%; m.p. >300 °C; M.wt.: 392.5; Anal. Calc. for C_16_H_15_CuN_3_O_5_: C, 48.91; H, 3.82; Cu, 16.17; N, 10.70%. Found: C, 49.02; H, 3.59; Cu, 16.03; N, 10.48%. Main IR peaks (KBr, cm^−1^): υ(NH) 3191; υ(C=O) 1673; υ(C=N)-; υ(Ar-OH) 1138; ʋ(Ar–O–C_aliphatic_) 1244; ʋ_as_(CH_3_COO^−^) 1529; ʋ_s_(CH_3_COO^−^) 1434. The complex is soluble in pyridine and is partially soluble in ethanol, methanol, acetonitrile, acetone, DMF and DMSO.

*Synthesis of the complex [Cu(L)(NO_3_)]·H_2_O* (**3**). Complex **3** was prepared in a similar fashion as complex **1**, using Cu(NO_3_)_2_·3H_2_O (0.242 g, 1 mmol). Brown solid. Yield: 79%; m.p. >300 °C; M.wt.: 413.5; Anal. Calc. for C_14_H_14_CuN_4_O_7_: C, 40.62; H, 3.38; Cu, 15.35; N, 13.54%. Found: C, 40.78; H, 3.21; Cu, 15.17; N, 13.32%. Main IR peaks (KBr, cm^−1^): υ(NH) 3196; υ(C=O) 1677; υ(C=N)-; υ(Ar-OH)-; ʋ(Ar–O–C_aliphatic_) 1244; ʋ_5_ (NO_3_^−^) 1530; ʋ_1_ (NO_3_^−^) 1214; ʋ_2_ (NO_3_^−^) 1032. The complex is soluble in pyridine and is partially soluble in ethanol, methanol, acetonitrile, acetone, DMF and DMSO.

*Synthesis of the complex [Cu(L)(ClO_4_)]·H_2_O* (**4**). Complex **4** was prepared in a fashion similar to complex **1**, using Cu(ClO_4_)_2_·6H_2_O (0.370 g, 1 mmol). Reddish brown solid. Yield: 84%; m.p. >300 °C; M.wt.: 451; Anal. Calc. for C_14_H_14_CuN_3_O_8_Cl: C, 37.25; H, 3.10; Cu, 14.07; N, 9.31%. Found: C, 37.49; H, 2.86; Cu, 13.91; N, 9.14%. Main IR peaks (KBr, cm^−1^): υ(NH) 3194; υ(C=O) 1678; υ(C=N)-; υ(Ar-OH) 1147; ʋ(Ar–O–C_aliphatic_) 1248; ʋ_3_(ClO_4_^−^) 1115; ʋ_4_(ClO_4_^−^) 1083. The complex is soluble in pyridine and is partially soluble in ethanol, methanol, acetonitrile, acetone, DMF and DMSO.

*Synthesis of the complex [Co(L)_2_]* (**5**). Complex **5** was prepared in a way similar to complex **1**, using CoCl_2_·6H_2_O (0.238 g, 1 mmol). Brown solid. Yield: 83%; m.p. >300 °C; M.wt.: 599; Anal. Calc. for C_28_H_24_CoN_6_O_6_: C, 56.09; H, 4.00; N, 14.02%. Found: C, 56.23; H, 3.87; N, 13.89%. Main IR peaks (KBr, cm^−1^): υ(NH) 3190; υ(C=O) 1672; υ(C=N)-; υ(Ar-OH) 1141; ʋ(Ar–O–C_aliphatic_) 1243. The complex is soluble in pyridine, DMF and DMSO, and is partially soluble in ethanol, methanol, acetonitrile, acetone.

*Synthesis of the complex [Zn(L)_2_]* (**6**). A chloroform solution (15 mL) of the ligand **HL** (0.271 g, 1 mmoL) was added to ZnCl_2_ (0.136 g, 1 mmoL) dissolved in distilled water (10 mL). The resulting solution was refluxed for 3 h. The yellow-colored solid was filtered, washed with hot water, methanol, ether and then dried in vacuo. Yield: 79%; m.p. >300 °C; M.wt.: 605.4; Anal. Calc. for C_28_H_24_ZnN_6_O_6_: C, 55.50; H, 3.96; N, 13.87%. Found: C, 55.69; H, 3.75; N, 13.60%. Main IR peaks (KBr, cm^−1^): υ(NH) 3192; υ(C=O) 1669; υ(C=N)-; υ(Ar-OH) 1143; ʋ(Ar–O–C_aliphatic_) 1250. The complex is soluble in pyridine, DMF and DMSO, and is partially soluble in ethanol, methanol, acetonitrile, acetone.

*Synthesis of the complex [Ni(L)(Cl)]* (**7**). To NiCl_2_·6H_2_O (0.238 g, 1 mmol) dissolved in 15 mL methanol ligand **HL** (0.271 g, 1 mmol) dissolved in 15 mL methanol was added and stirred at 50 °C for 5 h. The mixture was brought to pH 8 using a methanolic solution of KOH. The brown-colored precipitate was filtered, washed with methanol followed by ether and dried in vacuo. Yield: 81%; m.p. >300 °C; M.wt.: 364.2; Anal. Calc. for C_14_H_12_NiN_3_O_3_Cl: C, 46.12; H, 3.29; N, 11.53%. Found: C, 46.37; H, 3.18; N, 11.34%. Main IR peaks (KBr, cm^−1^): υ(NH) 3195; υ(C=O) 1672; υ(C=N)-; υ(Ar-OH)-; ʋ(Ar–O–C_aliphatic_) 1246. The complex is soluble in methanol, pyridine, DMF and DMSO, and is partially soluble in acetonitrile, acetone.

*Synthesis of the complex [Pt(L)(Cl)]* (**8**). Complex **8** was synthesized by stirring a hot solution of **HL** (0.271 g, 1 mmol) in ethanol (20 mL) with K_2_[PtCl_4_] (0.415, 1 mmol) dissolved in 20 mL distilled water. The mixture was stirred for 6 h at room temperature. The precipitate was filtered, washed with hot water and ethanol followed by ether and dried in vacuo. Yield: 80%; m.p. >300 °C; M.wt.: 500.5; Anal. Calc. for C_14_H_12_PtN_3_O_3_Cl: C, 33.56; H, 2.39; N, 8.39%. Found: C, 33.73; H, 2. 26; N, 8.18%. Main IR peaks (KBr, cm^−1^): υ(NH) 3195; υ(C=O) 1675; υ(C=N)-; υ(Ar-OH)-; ʋ(Ar–O–C_aliphatic_) 1250. The complex is soluble in DMF and DMSO, and is partially soluble in ethanol, methanol, acetonitrile, acetone.

### 3.3. Solubility Tests

#### Materials and Method

Solubility assessment was conducted in compendial aqueous solutions prepared according to United States Pharmacopoeia USP39/NF34): hydrochloric acid 0.1 N pH 1.2, acetate buffer pH 4.5 and phosphate buffer 6.8. These buffer systems are routinely used for screening of drug dissolution testing and they simulate the pH values of proximal gastrointestinal regions. The standard samples were prepared by diluting the stock solution of 80 µg/mL in dimethyl sulfoxide (with 0.2% pyridine, *v*/*v*) with corresponding media. The reagents were of analytical grade and used as received.

The assay was performed using a SpectraMax Plus 84 Plate reader reader (Sunnyvale, CA, USA), Molecular Devices LLC (US), equiped with SoftMax software version 6.4.2 (Sunnyvale, CA, USA). Separate plates were loaded with standard solutions in nine concentrations (0.2 to 80 µg/mL). For evaluation of the aqueous solubility, the samples were prepared in triplicate by adding 10 µL of the stock solution to the wells loaded with 190 µL of each buffer, using MultiScreen HTS plates (PCF, code MSSLBPC, Merck Millipore, Darmstadt, Germany). After 30 minutes of incubation at 37 ± 0.5 °C under agitation (250 rpm, IKA KS 4000 ic control, IKA, Staufen, Germany), the plates were filtrated under vacuum using a Vac-Man 96 Vacuum manifold (Promega Corporation, Madison, WI, USA). The solutions were collected in V-bottom polypropylene 96-well microplates (Greiner Bio-One, Kremsmünster, Austria). Volumes of 100 µL were transferred to UV-Star 96-well microplate with flat bottom (Chimney well, Grained Bio-One) and diluted by addition of 100 µL of the corresponding medium. The absorbance corresponding to each well was determined at fixed wavelengths: 280, 300, 320, 340, 360 and 800 nm. The reduced value of absorbance, calculated by the sum of the values recorded at the six wavelengths, were used for the calibration curve and subsequent determination of concentration in each solubility sample.

### 3.4. Daphnia magna Toxicity Assay

#### 3.4.1. Materials and Methods

*Daphnia magna* Straus originated from a culture maintained parthenogenetically at ‘Carol Davila’ University (Department of Pharmaceutical Botany and Cell Biology) from 2012. Young organisms were selected according to their size and kept in fresh artificial medium for 24 h. The bioassay was performed according to the method described in the literature [55] with some modifications [56,57,58]. In brief, ten daphnids were inserted in cell culture 12-well plates, and the compounds were added in artificial medium in order to obtain solutions of concentrations in range of 1 to 500 µM (1, 5, 10, 50, 100 and 500 µM). The final test solutions contained up to 1% DMSO and had a final volume of 4 mL. A 1% solution of DMSO in artificial medium was used as a negative control; the ligand and the metal salts corresponding to each coordination complex were used as positive controls. Throughout the experiment, the daphnids were kept in a growth chamber (Sanyo MLR-351H; Sanyo, San Diego, CA, USA) at 25 ± 1 °C, using a 16 h photoperiod and 8 h of darkness. The number of surviving daphnids was counted after 24 h and 48 h. The experiment was performed in duplicate. The daphnids were considered dead only if they did not move their appendages for 30 s during observations.

#### 3.4.2. Statistical Analysis

Student’s *t*-test was applied in order to analyze the results obtained on two replicates. Statistical significance was established by the Student’s *t*-test at the level of *p* < 0.05. The lethality percentage (L %) was calculated and plotted against the logarithm of concentrations and the lethality-concentration curves were calculated using the least squares fit method. The lethal concentrations (LC_50_) which produce a L % value of 50 were determined by interpolation on lethality-concentration curves. The upper and lower limits of the 95% confidence interval (CI 95%) and the correlation coefficient (r^2^) were calculated. All calculations were performed using GraphPad Prism version 5.01 software (GraphPad Software, Inc., La Jolla, CA, USA).

### 3.5. Antibacterial and Antifungal Activity

The antibacterial activity of complexes and also of their prototype furacilin has been determined under liquid nutritive environment (2% of peptonate bullion (pH 7.0)) using the successive dilutions method. *Escherichia coli*, *Klebsiella pneumoniae*, and *Staphylococcus aureus* stems were used as reference culture for the in vitro experiment. The dissolution of studied substances in dimethylformamide, microorganism cultivation, suspension obtaining, determination of minimal inhibition concentration (MIC) and minimal bactericide concentration (MBC) have been carried out according to the method previously reported.

Antimycotic properties of the complexes were investigated in vitro on laboratory stem *Candida albicans*. The activity has been determined in a liquid Sabouraud nutritive environment (pH 6.8). The inoculates were prepared from fungi stems which were harvested over 3–7 days. Their concentration in suspension is (2–4) × 10^6^ colonies form unities in milliliter. Sowings for levures and micelles were incubated at 37 °C during 7 and 14 days, respectively.

### 3.6. Cytotoxic Activity

#### 3.6.1. Preparation of Tested Substances Solutions

Compounds **HL**, **1**, **5**, **6** and **8** were dissolved in dimethyl sulfoxide (DMSO; Sigma-Aldrich, Ayrshire, UK) and stored at 2–8 °C. Compounds **2**, **3**, **4** and **7** were dissolved in pyridine (Fluka, Munich, Germany). A stock solution of 10 mM in the proper solvents was prepared; as for the final concentration (10 µM), successive dilution of the stock solution with culture medium was made.

#### 3.6.2. Cell Culture

The breast cancer cell lines were cultured as follow: MCF-7 human breast cancer cell line (ATCC, Rockville, MD, USA) was cultured in EMEM (Sigma Aldrich, Munich, Germany) supplemented with 10% FCS (fetal bovine serum, PromoCell, Heidelberg, Germany), 1% penicillin-streptomycin (Pen/Strep, 10,000 IU/mL; PromoCell, Heidelberg, Germany), 1% nonessential amino acids and 1% glutamine (PromoCell, Heidelberg, Germany). SKBR-3 human breast cancer cell line was cultured in McCoy’s 5a Medium Modified (Sigma Aldrich, Munich, Germany) supplemented with 10% FCS (fetal bovine serum, PromoCell, Heidelberg, Germany), 1% penicillin-streptomycin (Pen/Strep, 10,000 IU/mL; PromoCell, Heidelberg, Germany). A375 human melanoma cell line was cultured in DMEM containing 15% FCS (fetal bovine serum, PromoCell, Heidelberg, Germany) and 1% penicillin-streptomycin (Pen/Strep, 10,000 IU/mL; PromoCell, Heidelberg, Germany). Cells were maintained at an atmosphere of 5% CO_2_ at 37 °C. NCI-H1573 lung adenocarcinoma cell line was cultured in RPMI-1640 Medium (ATCC, Rockville, MD, USA) supplemented with 10% FCS (fetal bovine serum, PromoCell, Heidelberg, Germany), 1% penicillin-streptomycin (Pen/Strep, 10,000 IU/mL; PromoCell, Heidelberg, Germany).

#### 3.6.3. Cell Viability Assay: Alamar Blue In Vitro Analysis

All tested cell lines were seeded onto a 96-well microplate (5000 cells/plate) and left overnight to attach to the bottom of the well. After 24 h, the amount of 150 μL of the tested substances dissolved into the culture medium was added and cells were incubated for 48 h. After the incubation period, a volume of 15 μL of the Alamar blue (BioSource, Camarillo, CA, USA) solution was added. After another 10 h of incubation at 37 °C, the samples were spectrophotometrically analyzed using a microplate reader. The wave lengths used were 570 nm and 600 nm, respectively. Wells with untreated cells were used as controls. The highest concentration of DMSO and pyridine (0.1%) used to prepare stock solutions was also tested, in order to identify the solvent activity. All measurements were performed in triplicate and data were presented as mean ± standard deviation.

## 4. Conclusions

New copper(II), cobalt(II), zinc(II), nickel(II) and platinum (II) complexes derived from *N*-isonicotinoyl-*N*′-(3-metoxy-2 hydroxybenzaldehyde)-hydrazine have been synthesized and characterized. The physico-chemical analyses confirmed the composition and the structures of the newly obtained complexes. In all the complexes, the hydrazone **HL** acts as mononegative tridentate around the metallic ion. The EPR (electron paramagnetic resonance) spectra of the copper(II) complexes **1**–**4** in dimethyl sulfoxide (DMSO) solution confirmed the new structures. The EPR parameters gǁ, g⊥, Aǁ and the energies of d–d transitions were used to evaluate the bonding parameters. The orbital reduction factors, α^2^ and β^2^ indicate the presence of in-plane π bonding for all complexes. The f values of complexes **1**–**4** indicate small distortion from planarity.

The ligand and the metal complexes **1**–**8** have been screened for their in vitro antimicrobial activity against *Escherichia coli*, *Klebsiella pneumoniae*, *Staphylococcus aureus* and *Candida albicans* strains and for their antiproliferative activity against MCF-7 and SKBR-3 human breast, A375 human melanoma, and NCI-H1573 lung adenocarcinoma cells.

The quantitative antimicrobial activity test results proved that both the ligand and complex combinations have specific antimicrobial activity, depending on the microbial species tested.

The copper complexes **2**–**4** manifest a significant improvement of the cytotoxic effect in all four cell lines tested, while complexes **1**, **6**, **7** and **8** acted in a different manner, depending on the cell type. The newly synthesized complexes are less toxic for *Daphnia magna* than the salts and even than the ligand **HL**. The copper complexes showed promising antiproliferative activity and low toxicity on *Daphnia magna*.

The biological property of these compounds can be explained on the basis of several factors involving type of donor atom present in ligands, the metal ion type and coordination geometry. In conclusion, our results may be useful in designing novel Cu(II) antimicrobial and antiproliferative agents.

## Figures and Tables

**Table 1:** Electronic spectral assignments (cm^−1^) of the complexes **1**–**5**, **7** and **8**.

Compound	Transitions d–d (cm^−1^)			µ_eff_ (BM)	Geometry
[Cu(L)(Cl)]·2H_2_O (1)	^2^B_1g_→^2^B_2g_	^2^B_1g_→^2^E_g_	^2^B_1g_→^2^A_1g_	1.44	Square planar
	14,810	19,160	-		
[Cu(L)(CH_3_COO)] (2)	^2^B_1g_→^2^B_2g_	^2^B_1g_→^2^E_g_	^2^B_1g_→^2^A_1g_	1.73	Square pyramidal
	14,700	21,500	-		
[Cu(L)(NO_3_)]·H_2_O (3)	^2^B_1g_→^2^B_2g_	^2^B_1g_→^2^E_g_	^2^B_1g_→^2^A_1g_	1.78	Square pyramidal
	14,810	20,400	-		
[Cu(L)(ClO_4_)]·H_2_O (4)	^2^B_1g_→^2^B_2g_	^2^B_1g_→^2^E_g_	^2^B_1g_→^2^A_1g_	1.56	Square planar
	14,700	19,650	-		
[Co(L)_2_] (5)	^4^T_1g_(F)→^4^T_2g_(P)	^4^T_1g_(F)→^4^A_2g_(F)	^4^T_1g_(F)→^4^T_1g_(P)	4.60	Octahedral
	9660	-	17,390		
[Ni(L)(Cl)] (7)	^3^A_2g_(F)→^3^T_2g_(F)	^3^A_2g_(F)→^3^T_1g_(F)	^3^A_2g_(F)→^3^T_1g_(P)	3.46	Square-planar
	-	11,420	15,620		
[Pt(L)(Cl)] (8)	^1^A_1g_→^1^B_1g_	^1^A_1g_→^1^A_2g_	^1^A_1g_→^1^E_g_	*	Square-planar
		23,260			

* diamagnetic.

**Table 2:** EPR spectral parameters of the copper(II) complexes **1**–**4**.

	1	2	3	4
**Polycrystalline (298 K)**				
**g_//_**	2.249	2.210	2.170	2.251
**g⊥**	2.061	2.046	2.045	2.058
**DMSO (77 K)**				
**g_//_**	2.192	2.202	2.221	2.207
**g⊥**	2.045	2.043	2.068	2.052
**A_//_**	177	173	178	181
**α^2^**	0.739	0.737	0.781	0.768
**β^2^**	0.880	0.902	0.779	0.876
**δ^2^**	0.950	0.985	0.980	0.998
**K_//_**	0.651	0.665	0.608	0.674
**K⊥**	0.702	0.726	0.766	0.767

**Table 3:** The solubility of compound in compendial media (µg/mL, mean ± standard deviation, *n* = 3).

	Media	Hydrochloric Acid 0.1 N (pH = 1.2)	Acetate Buffer (pH = 4.5)	Phosphate Buffer (pH = 6.8)
Compound				
**HL**		2.02 ± 0.02	3.49 ± 0.41	0.25 ± 0.02
[Cu(L)(Cl)]·2H_2_O (**1**)		2.34 ± 0.28	1.89 ± 0.20	<0.2
[Cu(L)(CH_3_COO)] (**2**)		1.75 ± 0.54	1.27 ± 0.06	<0.2
[Cu(L)(NO_3_)]·H_2_O (**3**)		0.92 ± 0.11	1.69 ± 0.18	<0.2
[Cu(L)(ClO_4_)]·H_2_O (**4**)		0.82 ± 0.6	1.67 ± 0.28	<0.2
[Co(L)_2_] (**5**)		<0.2	1.62 ± 0.72	<0.2
[Zn(L)_2_] (**6**)		1.65 ± 0.06	1.56 ± 0.82	<0.2
[Ni(L)(Cl)] (**7**)		2.26 ± 0.44	1.91 ± 0.26	<0.2
[Pt(L)(Cl)] (**8**)		1.33 ± 0.05	2.49 ± 0.09	<0.2

**Table 4:** Toxicity on *Daphnia magna* of **HL**, metal salts and newly synthesized complexes.

Compound	Incubation Period					
	24 h	48 h	24 h	48 h	24 h	48 h
	LC_50_ (µM)		CI 95% of LC_50_ (µM)		Goodness of Fit (r^2^)	
**HL**	53.09	52.60	ND*	ND*	0.9918	0.9849
[Cu(L)(Cl)]·2H_2_O (**1**)	393.80	69.53	374.4–414.2	33.96–142.4	0.9966	0.8159
CuCl_2_	4.23	0.97	3.709–4.815	ND**	0.9890	0.9302
[Cu(L)(CH_3_COO)] (**2**)	69.59	13.09	44.11–109.8	8.89–19.26	0.9021	0.9389
Cu(CH_3_COO)_2_	9.68	0.44	ND**	ND**	0.9980	ND*
[Cu(L)(NO_3_)]·H_2_O (**3**)	316.90	5.61	2.19–2.81	0.51–0.98	0.7842	0.8742
Cu(NO_3_)_2_	7.43	1.39	6.04–9.15	1.08–1.78	0.9586	0.9446
[Cu(L)(ClO_4_)]·H_2_O (**4**)	ND*	ND*	ND**	ND*	ND*	ND*
Cu(ClO_4_)_2_	0.39	0.31	2.34–3.76	0.99–1.22	0.9777	0.9794
[Co(L)_2_] (**5**)	278.20	2.38	166.4–465.4	1.629–3.489	0.8655	0.9145
CoCl_2_	205.20	31.37	158.4–265.8	17.06–57.70	0.9717	0.876
[Zn(L)_2_] (**6**)	33.91	0.84	23.27–49.4	0.33–2.12	0.9452	0.7553
ZnCl_2_	15.39	0.89	9.42–25.15	0.79–1.02	0.9190	0.9691
[Ni(L)(Cl)] (**7**)	350.80	209.70	320.6–383.8	149.6–294.1	0.9935	0.9315
NiCl_2_	100.00	16.25	98.31–101.7	11.86–22.27	0.9827	0.9624
[Pt(L)(Cl)] (**8**)	468.60	78.19	400.6–548.3	57.44–106.5	0.9490	0.9546
K_2_PtCl_4_	27.70	5.63	21.86–35.11	3.953–8.012	0.9918	0.9364

ND*—not determined because maximum L% was 10% after 24 h and 35% after 48 h, respectively; ND**—not determined because CI 95% is very wide.

**Table 5:** Antibacterial and antifungal activities of complexes **1**–**8** as MIC ^a^/MBC ^b^ values (µg/mL).

Compounds	*E. coli* (G −)		*K. pneumoniae* (G −)		*S. aureus* (G +)		*C. albicans*	
	*MIC*	*MBC*	*MIC*	*MBC*	*MIC*	*MBC*	*MIC*	*MBC*
[Cu(L)(Cl)]·2H_2_O (**1**)	500	500	500	500	0.015	0.03	7	15
[Cu(L)(CH_3_COO)] (**2**)	500	500	500	500	0.015	0.015	125	250
[Cu(L)(NO_3_)]·H_2_O (**3**)	500	500	500	500	0.07	0.15	1.5	3
[Cu(L)(ClO_4_)]·H_2_O (**4**)	250	500	250	500	0.7	0.7	0.7	0.7
[Co(L)_2_] (**5**)	250	500	500	500	0.7	0.7	125	250
[Zn(L)_2_] (**6**)	125	250	250	250	63	125	125	125
[Ni(L)(Cl)] (**7**)	500	500	500	500	0.7	0.7	125	125
[Pt(L)(Cl)] (**8**)	125	250	250	500	0.7	0.7	63	63
Furacillinum	18.7	37.5	>300	>300	9.35	9.35		
Nystatine							80	80

*E. coli* (*Escherichia coli*, ATCC 25922); *K. pneumoniae* (*Klebsiella pneumoniae*, ATCC 31,488); *S. aureus* (*Staphylococcus aureus*, ATCC 25923); *C. albicans* (*Candida albicans*). ^a^ MIC–minimum inhibitory concentration; ^b^ MBC—minimum bactericide concentration. G(−): Gram-negative bacteria; G(+): Gram-positive bacteria.

## Supplementary Materials

Supplementary materials are available online.
