# Supplementary material for: Synthesis, Characterization, Antimicrobial and Antiproliferative Activity Evaluation of Cu(II), Co(II), Zn(II), Ni(II) and Pt(II) Complexes with Isoniazid-Derived Compound
Source: Molecules. 2017 Apr 19;22(4):650. doi: 10.3390/molecules22040650 (PMC6154339; doi:10.3390/molecules22040650)
Supplement: Supplementary file 1 [file molecules-22-00650-s001.pdf]

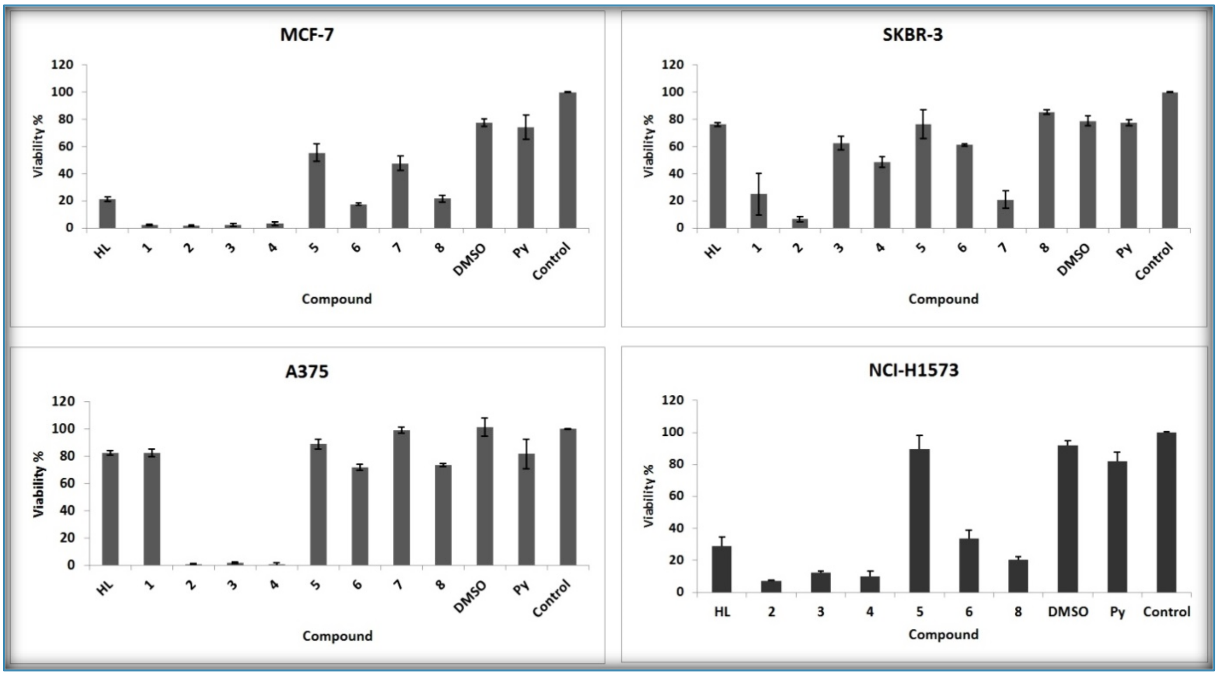

*Cell viability of the MCF-7, SKBR-3, A375 and NCI-H1573 cancer cells after 48 hours treatment with **HL** and the metal complexes*
