# Supplementary material for: Synthesis, Characterization, Antimicrobial and Antiproliferative Activity Evaluation of Cu(II), Co(II), Zn(II), Ni(II) and Pt(II) Complexes with Isoniazid-Derived Compound
Source: Molecules. 2017 Apr 19;22(4):650. doi: 10.3390/molecules22040650 (PMC6154339; doi:10.3390/molecules22040650)
Supplement: Supplementary file 2 [file molecules-22-00650-s002.pdf]

Supplementary information: Synthesis, Characterization, Antimicrobial and Antiproliferative Activity Evaluation of Cu(II), Co(II), Zn(II), Ni(II) and Pt(II) Complexes with Isoniazid-Derived Compound

Elena Pahontu *, Diana-Carolina Ilies *, Sergiu Shova, Camelia Oprean, Virgil Paunescu, Octavian Tudorel Olaru, Flavian Stefan Radulescu, Aurelian Gulea, Tudor Rosu, Doina Draganescu

Table S1. Crystal data and summary of intensity data collectionand structure refinement for **HL**

Compound	HL
Empirical formula	$C_{14}H_{13}N_3O_3$
Formula weight	271.27
Temperature (K)	200
Crystal system	monoclinic
Space group	$P2_1/n$
a (Å)	7.5757(5)
b (Å)	16.2389(7)
c (Å)	10.8065(7)
α (°)	90
β (°)	109.973(6)
γ (°)	90
V(Å^3)	1249.46(13)
Z	4
$D_{calc.}$ (g/cm^3)	1.442
μ (mm^{-1})	0.104
F(000)	568.0
Crystal size (mm)	0.45 × 0.2 × 0.2
θmin, θmax (°)	4.73 to 50.052
Limiting indices	$-9 \le h \le 8$<br>$-19 \le k \le 18$<br>$-12 \le l \le 12$
Reflections collected / unique	6626 / 2197<br>[R(int) = 0.0242]
Data/restraints/parameters	2197/0/183
Goodness-of-fit on F^2	1.064
Final R_1, wR_2 [I>2sigma(I)]	0.0397, 0.0941
R_1, wR_2 (all data)	0.0514, 0.1020
Largest diff. peak/ hole (eÅ^{-3})	0.20/-0.22

Table S2. FAB mass spectral data of complexes **1 - 8**.

Molecular formula	Mw (g/mol)	Molecular ion peak $[M]^+$	The peaks due to complex fragmentation			
$[Cu(L)(Cl)]\cdot 2H_2O$ (**1**)	405	407.1	218.2	249.9	302.3	388.1
$[Cu(L)(CH_3COO)]$ (**2**)	392.5	394.9	219.0	302.3	333.1	356.5
$[Cu(L)(NO_3)]\cdot H_2O$ (**3**)	413.5	411.1	195.0	241.0	302.4	333.1
$[Cu(L)(ClO_4)]\cdot H_2O$ (**4**)	451	452.2	225.2	302.3	321.5	352.2
$[Co(L)_2]$ (**5**)	599	604.0	333.1	452.1	512.5	568.6
$[Zn(L)_2]$ (**6**)	605.4	605.1	195.1	218.2	374.0	567.2
$[Ni(L)(Cl)]$ (**7**)	364.2	365.1	232.7	251.0	296.9	343.9
$[Pt(L)(Cl)]$ (**8**)	500.5	501.2	241.0	294.1	330.5	452.1

Table S3. Cell viability of the MCF-7, SKBR-3, A375 and NCI-H1573 cancer cells after 48 hours treatment with **HL** and the complexes.

		**HL**	**1**	**2**	**3**	**4**	**5**	**6**	**7**	**8**	**DMSO**	**Py**	**Control**
**MCF-7**	Viability %	21.53	2.47	2.10	2.44	3.43	55.61	17.72	47.75	21.71	77.79	74.39	100.00
	STD	1.83	0.70	0.65	1.06	1.57	6.57	1.01	5.19	2.37	2.89	8.87	0.20
**SKBR-3**	Viability %	76.26	25.24	6.88	62.65	48.72	76.68	61.43	21.11	85.27	78.90	77.51	100.00
	STD	1.40	15.30	1.99	5.01	3.88	10.59	0.85	6.53	1.66	3.58	2.12	0.20
**A375**	Viability %	82.43	84.43	0.73	1.59	0.70	88.96	71.66	98.97	73.45	101.48	81.57	100.00
	STD	1.90	2.57	0.27	0.53	1.02	3.60	2.15	2.14	1.03	6.67	10.71	0.40
**NCI-H1573**	Viability %	29.16		7.22	12.14	10.05	89.79	33.77		20.34	91.88	81.84	100.00
	STD	5.26		0.39	0.99	3.45	8.11	5.10		2.08	2.85	5.78	0.30

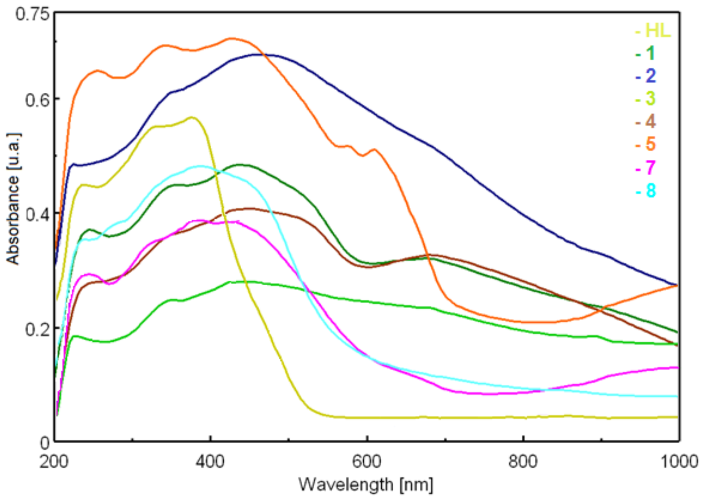

Figure S1. Electronic spectra of the ligands **HL** and metal complexes **1 – 5**, **7**, **8.**

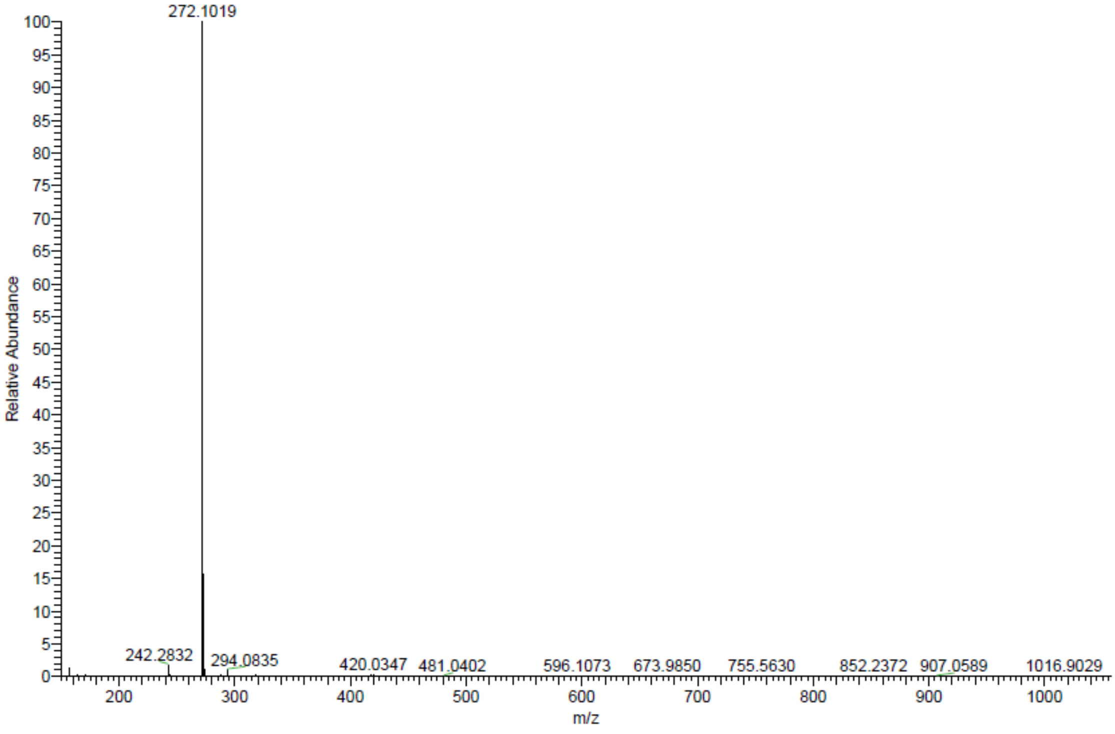

Figure S2. Mass spectrum of ligand **HL**

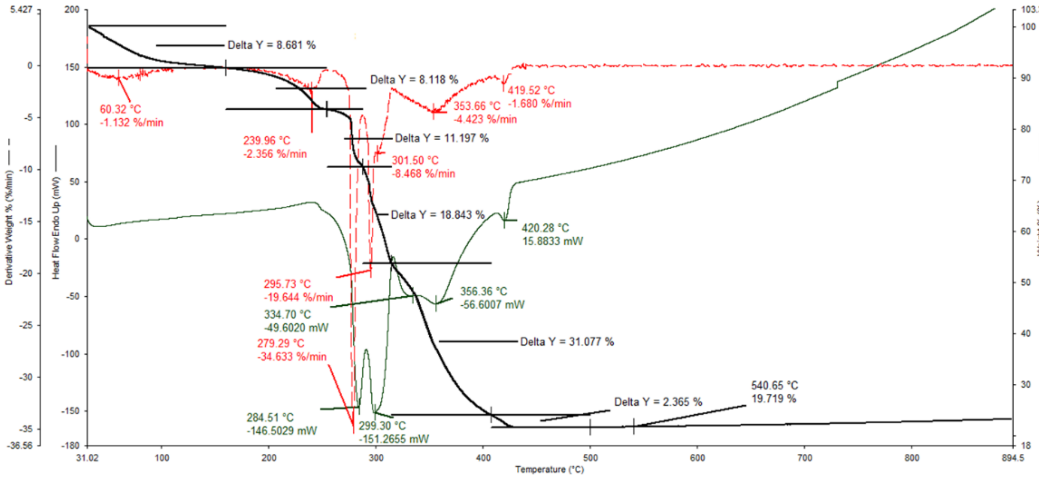

(a)

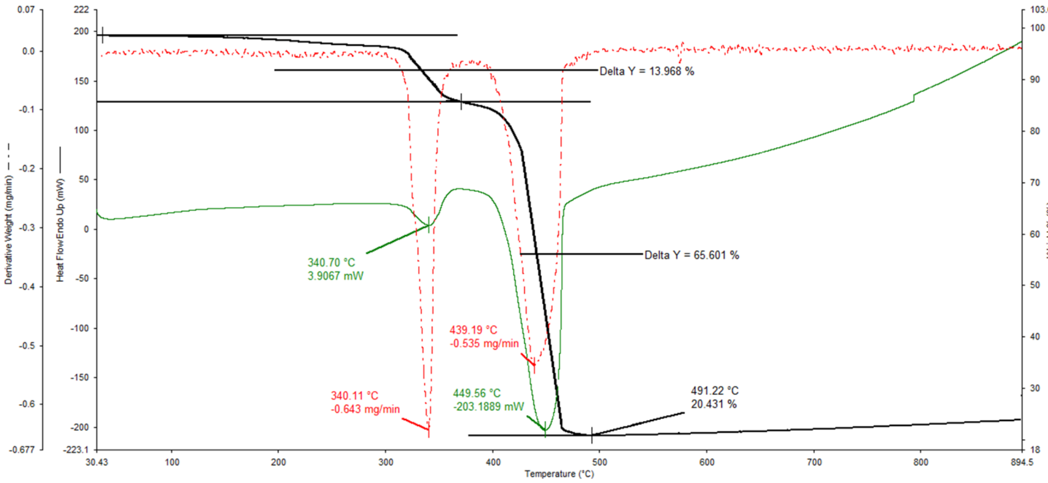

(b)

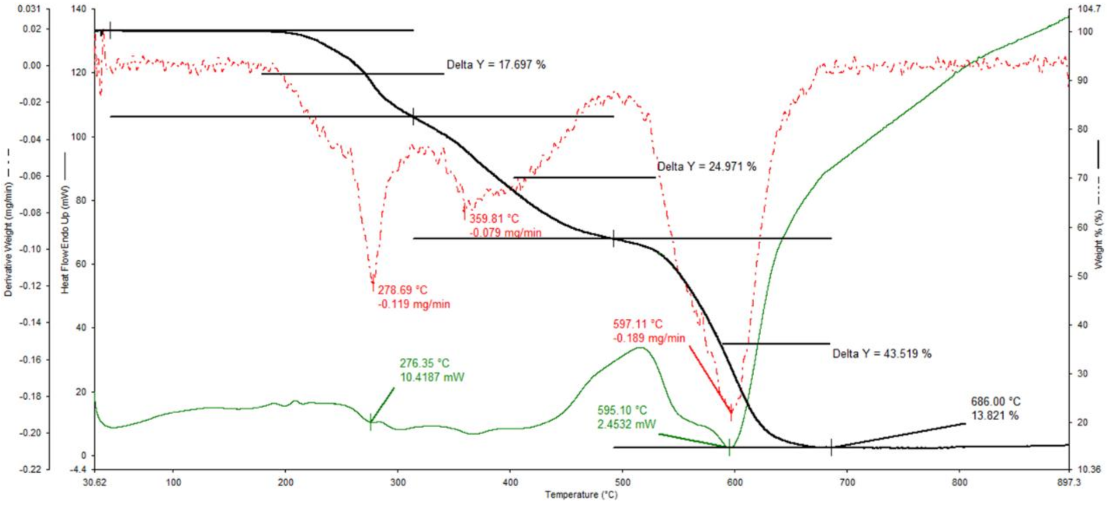

(c)

Figure S3. Thermogravimetric analysis of complexes (a)**1**, (b)**2** and (c)**6**.

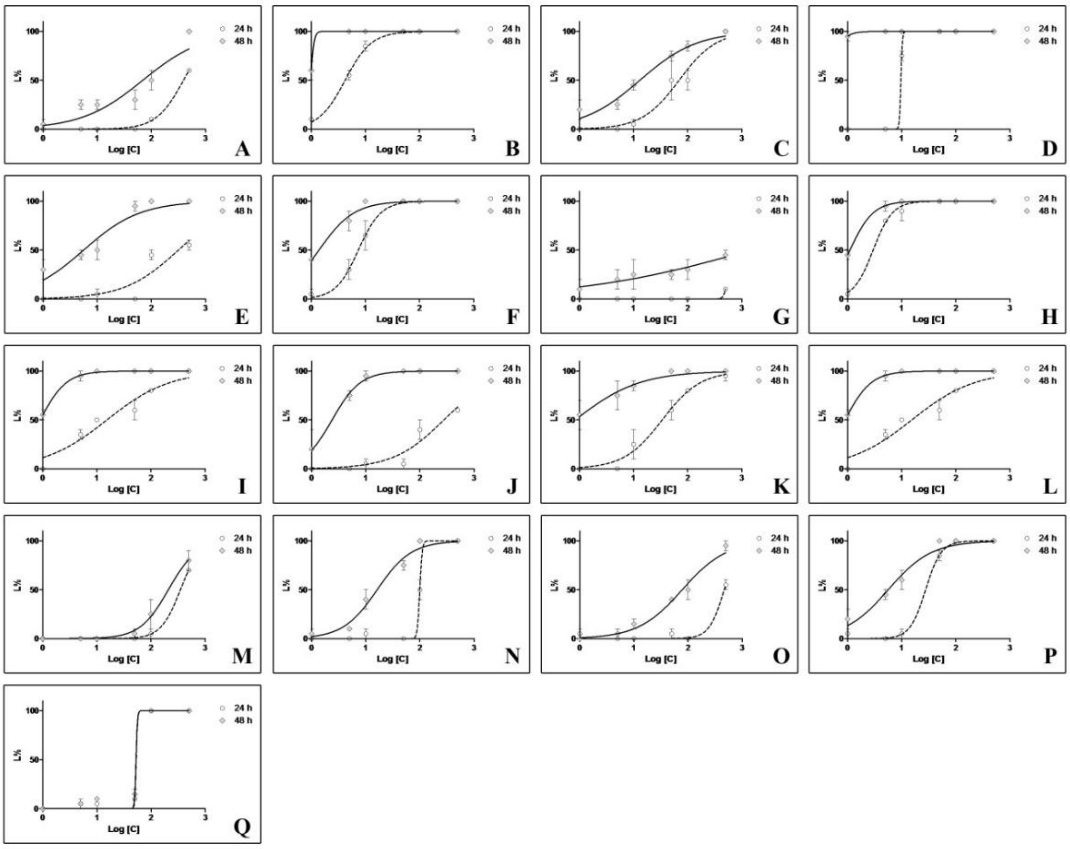

Figure S4. Concentration-lethality curves for toxicity assessment on *Daphnia magna* of (A) **1**, (B) $CuCl_2$, (C) **2**, (D) $Cu(CH_3COO)_2$, (E) **3**, (F) $Cu(NO_3)_2$, (G) **4**, (H) $Cu(ClO_4)_2$, (I) **5**, (J) $CoCl_2$, (K) **6**, (L) $ZnCl_2$, (M) **7**, (N) $NiCl_2$, (O) **8**, (P) K_2PtCl_4 and (Q) **HL**.
